# Development of endosperm transfer cells in barley

**DOI:** 10.3389/fpls.2014.00108

**Published:** 2014-03-26

**Authors:** Johannes Thiel

**Affiliations:** Department of Molecular Genetics, Leibniz Institute of Plant Genetics and Crop Plant Research (IPK)Gatersleben, Germany

**Keywords:** barley seed, endosperm transfer cells, cell wall ingrowths, tissue-specific analysis, nutrient transport, TCS, ABA, ethylene

## Abstract

Endosperm transfer cells (ETCs) are positioned at the intersection of maternal and filial tissues in seeds of cereals and represent a bottleneck for apoplasmic transport of assimilates into the endosperm. Endosperm cellularization starts at the maternal-filial boundary and generates the highly specialized ETCs. During differentiation barley ETCs develop characteristic flange-like wall ingrowths to facilitate effective nutrient transfer. A comprehensive morphological analysis depicted distinct developmental time points in establishment of transfer cell (TC) morphology and revealed intracellular changes possibly associated with cell wall metabolism. Embedded inside the grain, ETCs are barely accessible by manual preparation. To get tissue-specific information about ETC specification and differentiation, laser microdissection (LM)-based methods were used for transcript and metabolite profiling. Transcriptome analysis of ETCs at different developmental stages by microarrays indicated activated gene expression programs related to control of cell proliferation and cell shape, cell wall and carbohydrate metabolism reflecting the morphological changes during early ETC development. Transporter genes reveal distinct expression patterns suggesting a switch from active to passive modes of nutrient uptake with the onset of grain filling. Tissue-specific RNA-seq of the differentiating ETC region from the syncytial stage until functionality in nutrient transfer identified a high number of novel transcripts putatively involved in ETC differentiation. An essential role for two-component signaling (TCS) pathways in ETC development of barley emerged from this analysis. Correlative data provide evidence for abscisic acid and ethylene influences on ETC differentiation and hint at a crosstalk between hormone signal transduction and TCS phosphorelays. Collectively, the data expose a comprehensive view on ETC development, associated pathways and identified candidate genes for ETC specification.

## INTRODUCTION

Transfer cells (TCs) occur ubiquitously in all taxonomic groups of the plant kingdom as well as in algae and fungi. TCs represent highly specialized cells modified to facilitate enhanced transport capacities of nutrients. Unique characteristics of these cells are thickened cell walls and massive, localized wall ingrowths to enrich the membrane surface for transport processes. In general, TCs are located at strategically important positions for nutrient acquisition and exchange, such as symplasmic/apoplasmic interfaces in vascular tissues and seeds, reproductive and secretory organs and at contact points of plant/symbiotic and plant/parasitic interactions. In the last decade, TCs in seeds have been the object of intensive investigation due to their pivotal role in feeding the new generation and the impact on seed filling. Developing seeds are sink tissues depending on nutrient supply from vegetative tissues, accordingly efficient assimilate transfer into seeds is yield-determining and of high agronomical interest. In the developing barley grain, incoming assimilates are released from the maternal grain part by the nucellar projection (NP), which is responsible for transfer but also for interconversion of assimilates, especially amino acids (Thiel et al., 2009). Released assimilates are taken up by endosperm transfer cells (ETCs) and supplied to the endosperm. ETCs are positioned at the maternal-filial boundary of grains and might also play an essential role in communication of maternal and filial tissues by transmitting signals into the endosperm (Thiel et al., 2008).

Anatomy of TCs in seeds can be distinguished by two principle types of cell wall ingrowth architecture, the “flange” or “reticulate” type. A prominent example for the reticulate wall ingrowth-type is represented in epidermal TCs of *Vicia faba* cotyledons. Reticulate wall ingrowths are initiated as discrete papillar projections that appear as randomly located depositions on the primary cell wall. Further repetitions of branching and fusion with neighboring ingrowths result in the formation of a multi-layered, fenestrated wall ingrowth labyrinth (Talbot et al., 2001; McCurdy et al., 2008). Flange wall ingrowths are deposited as parallel ribs or bands of cell wall material emerging from the primary cell wall. By affiliation of adjacent ribs -predominantly toward the basal part of the cell- a dense network of flanges is created. This morphology is found in basal endosperm transfer cells (BETCs) of maize kernels (Talbot et al., 2002; Zheng and Wang, 2010) and ETCs in wheat and barley (Zheng and Wang, 2011; Thiel et al., 2012a). In addition to these peculiar anatomical differences between TCs observed in legume and cereal seeds, the genetic origin of the cells is fundamentally different. Legume TCs originate from the diploid embryo by a trans-differentiation process converting abaxial epidermal cells of the cotyledons into TCs (Offler et al., 1997). This process of “re-differentiation” of cells is deemed to be part of the developmental program of several types of TCs or is induced by abiotic and/or biotic stresses. ETCs of cereal grains are part of the triploid endosperm which is composed of four different cell types: starchy endosperm, embryo-surrounding region, aleurone and TCs. After fertilization of the central cell of the megagametophyte by the second male gamete, endosperm development of barley starts with divisions of nuclei without building of cell walls resulting in the formation of the endosperm coenocyte. Cell fate specification occurs already in the endosperm coenocyte (Olsen, 2001) and is proposed to be determined by positional signaling (Gruis et al., 2006). Endosperm cellularization is accompanied by the differentiation of the NP, the part of the nucellus facing the main vascular bundle. Cellularization of the endosperm starts around 3–4 days after flowering (DAF) in the outermost cell row adjacent to NP generating the highly specialized ETCs whereas the other cells in peripheral positions assume aleurone cell fate later on (6/7 DAF). This raises the question which genetic regulators give rise for the early specification of this distinct region of the syncytium which later on differentiates to ETCs while other endosperm cells differentiate to aleurone, subaleurone and starchy endosperm. The *END1* gene of barley and its wheat ortholog has been associated to the specification of the ETC region due to the specific expression in the coenocytic nuclei which is a prerequisite for development of ETCs (Doan et al., 1996). Another molecular marker inducing TC identity has been found in maize BETCs by Gomez et al. (2002). ZmMRP-1 is a MYB-related R1-type transcription factor and has been shown to transactivate the promoters of *BETL1* (Gomez et al., 2002), Meg1 (Gutierrez-Marcos et al., 2004) as well as *ZmTCRR1* (Muñiz et al., 2006) genes, which are also specifically expressed in BETCs. The imprinted *Maternally expressed gene 1* (*ZmMeg1*) is located at the plasma membrane and extracellular matrix of ETCs and has been shown to play a key role in specification and differentiation of BETCs. *Meg1*-knockdown resulted in impaired BETC development with diminished cell wall ingrowths and clearly affected nutrient uptake, sucrose partioning and seed biomass (Gutierrez-Marcos et al., 2004; Costa et al., 2012). As mentioned, other genes specifically expressed in maize BETCs encode for members of the small, cystein-rich BETL family (BETL1-4), two type-A response regulators (ZmTCRR1,-2; Muñiz et al., 2006, 2010) and for the *Mn1*-encoded cell wall invertase 2 (INCW2; Cheng et al., 1996). However, these genes are predominantly expressed during the mid-term endosperm development (8-16 DAP) and might therefore play a role in structural specification of TCs (i.e., establishment of wall ingrowths) rather than in acquisition of cell identity. Remarkably, the expression of *ZmMRP-1* is induced by sugars, most effectively by glucose (Barrero et al., 2009), which might be attributed to INCW2 activity. The phytohormones auxin and ethylene have been shown to function as important compounds determining TC differentiation. Dibley et al. (2009) uncovered a role for auxin and ethylene signaling in TC induction and development by using an *in vitro* culture system to selectively induce trans-differentiation to a TC morphology in *V. faba* cotyledons. The expression of ethylene biosynthesis genes and ethylene signaling elements in tight correlation with ROS signaling might play an inductive role in formation of polarized cell wall ingrowths in *Vicia* cotyledons (Zhou et al., 2010; Adriunas et al., 2011). A role for ethylene in ETC differentiation in barley seeds was elucidated by LM-based transcript profiling of ETCs and cells of the NP (Thiel et al., 2008), which revealed a preferential expression of enzymes associated with ethylene biosynthesis and catabolism as well as several AP2/EREBP transcription factors in ETCs. This is in agreement with the finding that direct application of the ethylene precursor 1-aminocyclopropane-1-carboxylic acid (ACC) increased the number of cells forming wall ingrowths during TC formation in tomato roots (Schikora and Schmidt, 2002).

However, the underlying molecular mechanisms determining acquisition of TC fate and further development are poorly understood. Subsequently, the elucidation of signals activating key regulators directing ETC specification in the syncytial endosperm and further differentiation to achieve characteristic TC anatomy would be a challenging task toward a deeper understanding of these specialized plant cells.

This article reviews results from tissue-specific transcriptome and metabolome analyses which have been performed by using LM-based techniques for the isolation of this specific cell type. Transcriptionally activated pathways, regulatory networks as well as new modes of signal transduction potentially implicated in barley ETC differentiation are highlighted in the article.

## HISTOLOGICAL ANALYSIS OF BARLEY ENDOSPERM TRANSFER CELLS DEPICTS DISTINCT TIME POINTS IN ESTABLISHING TRANSFER CELL MORPHOLOGY

**FIGURE 1:**
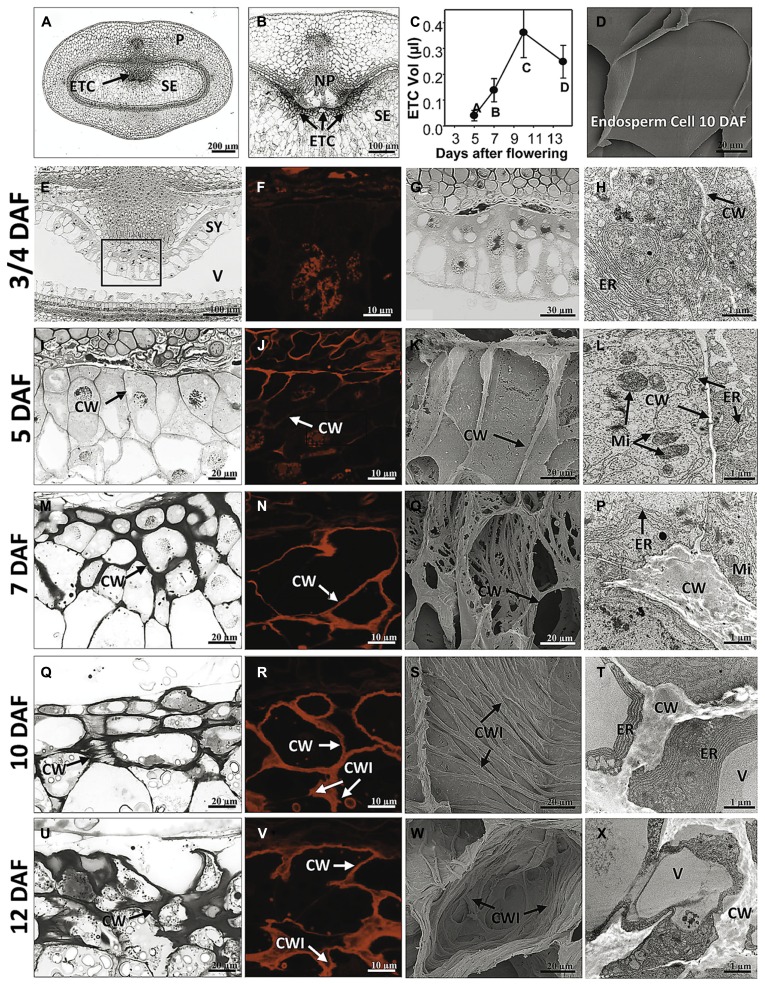
**Morphological and ultrastructural analysis of barley ETC development.** Light microscopy **(E,G,I,M,Q,U)**, DIC fluorescence microscopy **(F,J,N,R,V)**, scanning electron **(K,O,S,W)** and transmission electron microscopy **(H,L,P,T,X)** images show ETC differentiation from cellularization (3/4 DAF) until the establishment of transfer cell structure (12 DAF). **(A)** Median cross section of a barley grain at 7 DAF depicts grain tissues and **(B)** magnification shows the transfer region of barley grains. **(C)** Volume of ETCs from 5 to 14 DAF, **(D)** scanning electron picture of an endosperm cell at 10 DAF. At 3/4 DAF, cellularization in the region of the syncytium facing the nucellar projection has just started **(E,G)**. Cells contain a dense cytoplasm, rich in organelles of the endomembrane secretory system, and segments of cell walls are apparent but do not border complete cells (arrow, **H**). At 5 DAF, cellularization of the ETC region is completed; up to three rows of cells facing the NP are apparently different from the other cells of the endosperm **(I)**. Cell walls appear subtle and thin; branching and deposition of cell wall ingrowths are not visible **(K)**. Cells show a strong compartmentation and multiple mitochondria predominantly adjacent to the cell wall (arrows, **L**). At 7 DAF, cell walls are clearly thickened **(M)** and first branches of ribbed cell wall depositions covering the inner surface of walls can be detected **(O)**. At 10 DAF, area of cell walls further increase and cells are completely lined by massive walls **(Q,R)**. Cell walls show parallel rib-shaped depositions characteristic for the flange-type ingrowths **(S)**. ER appeared to be constantly striped and nestled to the thickened walls **(T)**. At 12 DAF, cell wall and ingrowths emerge asymmetrically and cover the main part of the cell **(U–X)**. The cytoplasm is reduced compared to earlier stages **(X)**. CW, cell wall; CWI, CW ingrowths; ER, endoplasmic reticulum; Mi, mitochondria; ETC, endosperm transfer cells; NP, nucellar projection; SE, starchy endosperm; SY, syncytium; V, vacuole (images modified after Thiel et al., 2012a).

Morphogenesis of barley ETCs from initial steps (just before/at cellularization) until establishment of TC structure (3/4 to 12 DAF) was analyzed by light, scanning electron and transmission electron microscopy. An overview about barley grain tissues and a magnification of the crease region is given in **Figures 1A,B**. At 3/4 DAF, the filial grain part is composed of the endosperm coenocyte, a seam of cytoplasm containing free nuclei, surrounding the large endosperm vacuole (**Figure 1E**). Cellularization starts in the region of the syncytium facing the NP whereas in the dorsal part of the syncytium cellularization has not yet been initiated. First smooth and thin cell walls are visible in the emerging cell row, dividing nuclei indicate high mitotic activity in the ETC region (**Figures 1F,G**). The cells contain a dense cytoplasm rich in organelles of the endomembrane secretory system, like the endoplasmatic reticulum (ER), dictyosomes and secretory vesicles. Cellularization has just started and segments of cell walls are apparent but seem not to border complete cells (**Figure 1H**). At 5 DAF, cellularization of the ETC region is completed. Up to three rows of cells facing the NP are apparently different from the other cells of the endosperm (**Figure 1I**). They are characterized by a rounded shape, smaller size, and dense cytoplasm with small vacuoles. Cell walls appear subtle and thin; branching and deposition of cell wall ingrowths are not visible at 5 DAF (**Figure 1K**). The slender nature of wall anatomy is supported by DIC fluorescence microscopy (**Figure 1J**) which was used as a method to visualize cell wall morphology by detecting the autofluorescence of cellulose-containing cell contents. As illustrated in further pictures (**Figures 1N,R,V**), the red autofluorescence reflects the changing cell wall architecture as observed by electron microscopy. Cells show a strong compartmentation with organelles of the secretory system (ER and dictyosomes) and multiple mitochondria predominantly adjacent to the cell wall (**Figure 1L**).

At 7 DAF, differences in the cell wall architecture between ETCs and endosperm cells became pronounced. Cell walls are clearly thickened preferentially in the direction toward the endosperm (**Figure 1M**). First branches of ribbed cell wall depositions covering the inner surface of walls can be detected (**Figure 1O**). Fusion of branched flange-like strands is predominantly visible in the basal part of the cell at the corners contacting adjacent cells and thereby indicating a differentiation gradient in the formation of wall ingrowths (**Figure 1P**). More generally, the structure of the intracellular compartments seems to change at 7 DAF: central vacuoles and deposition of small starch granules can be monitored as well as a strong orientation of mitochondria and banded ER to wall ingrowths. Presumably, this re-arrangement of cellular organization is related to the initiation of wall ingrowth formation. Areas of cell walls further increase during ongoing development of ETCs (10 DAF) and cells are completely lined by massive walls (**Figure 1R**). Cell walls show parallel rib-shaped projections fused at parallel protrusions (**Figure 1S**) which lead to the characteristic Y-shaped structure of flanges as visible in the TEM picture (**Figure 1T**). In contrast, cells of the starchy endosperm -located only some rows below- show a glossy surface without any wall depositions (**Figure 1D**). ETCs obtain a more rectangular shape and starch grains are visible in the second and third layer of ETCs (**Figure 1Q**). ER appeared to be constantly striped and nestled to the thickened walls. At 12 DAF, cell wall and its ingrowths emerge asymmetrically and thereby, covering the main part of the cell (**Figures 1U–X**). The cytoplasm of ETCs is less dense and seems to be reduced compared to earlier stages (**Figure 1X**); fewer mitochondria and dictyosomes can be observed and the cell surface is dominated by a large vacuole and storage bodies (starch, lipids).

### ESTABLISHMENT OF TRANSFER CELL MORPHOLOGY PROCEEDS ENDOSPERM GROWTH

Growth behavior of ETCs and changes in size was determined using 3D histological images during barley grain development (5–14 DAF) by calculating the geometric expansions in transverse and longitudinal directions (Bollenbeck and Seiffert, 2008). The volume of ETCs increases 9-fold from 5 to 10 DAF but decreases thereafter around 30% at 14 DAF (**Figure 1C**). Interestingly, the volume increment of ETCs proceeds that of the endosperm which shows a strong augmentation between 10 and 14 DAF (Thiel et al., 2012a). Thus, it can be concluded that ETCs have to be fully developed before the endosperm growth rises exponentially. This clearly supports the concept that ETCs represent a kind of bottleneck for assimilate transfer into the filial grain parts and suggests that grain filling is tightly related to the developmental status of ETCs.

## TISSUE-SPECIFIC STUDIES UNRAVELED DEVELOPMENTAL PROGRAMS AND ASSOCIATED PATHWAYS DURING ENDOSPERM TRANSFER CELL DIFFERENTIATION

### EARLY ETC DIFFERENTIATION IS CHARACTERIZED BY TRANSCRIPTIONAL ACTIVITIES REGULATING CELL SHAPE AND RELATES INTENSIVE CELL WALL TO RESPIRATORY METABOLISM

Hidden inside the grain, ETCs are barely accessible by manual preparation. To get information about molecular processes occurring in ETCs, LM-assisted methods for transcript and metabolite analyses of barley grain tissues have been developed. Microarray analysis of ETCs at different stages of grain development (5–12 DAF) provided comprehensive information about transcriptionally activated pathways and underlying transcriptional networks determining ETC differentiation (Thiel et al., 2012a). Intriguingly, at the early stages of differentiation concurrent with the cellularization process a multitude of transcripts related to cell division, cell shape control, intracellular vesicle trafficking as well as cell wall biosynthesis and primary metabolism are preferentially expressed. Transcripts encoding cyclins, proteins controlling cell cycle and cell cycle-associated kinases as well as microtubule associated proteins, ß-tubulins and expansins indicate distinct cell proliferation and cell shape control at 5 DAF. Simultaneously more than 40 transcripts related to vesicle formation and vesicle transport are predominantly expressed at 5 and 7 DAF, among them, genes encoding kinesins, motor-related proteins, actins and tubulins. Two transcripts encoding Rab-GTPases are upregulated at 7 DAF which potentially bind intracellular vesicles to recruit motor proteins (Deneka et al., 2003).

V-type ATPases, autophagy-related proteins and vacuolar sorting proteins might be involved in endocytic and secretory trafficking. This reflects the dense accumulation of organelles of the endomembrane secretory system in the cytoplasm of early ETCs. Cell wall biosynthesis is predominantly stimulated at 5 DAF and to a less degree at 7 DAF. Transcription of several cellulose synthase-like and cellulose synthase genes accompanied by glucan synthases and transferases, enzymes involved in biosynthesis of mannose sugars, arabinoxylans and pectins indicate complex patterns in cell wall biosynthesis. Remarkably is the expression profile of genes involved in callose metabolism like 1,3-ß-glucan synthases/transferases and 1,3-ß-glucanases with a preferential expression at 5 DAF indicating a high turnover just after cellularization. In endosperm cells, callose has been shown to be involved in cell plate formation and early cell wall development (Brown et al., 1997) but the exact role in ETC differentiation remains unclear. Distinct transcripts related to primary metabolism are preferentially expressed at 5 and 7 DAF: high transcript amounts of enzymes related to mitochondrial activity (TCA cycle, respiration) and glycolysis particularly at 7 DAF suggest the demand for energy which is probably needed for ATP-consuming processes, such as cell wall biosynthesis and its further differentiation. The abundance of transcripts involved in carbohydrate/starch metabolism supports this assumption. Furthermore, multiple genes transcriptionally activated at 5 and 7 DAF are associated to amino acid catabolism and the adjustment of C:N balances (Thiel et al., 2012a). Taken together, the data set provides insights into transcriptional pathways that relate energy-generating metabolic activities and stimulated cell wall biosynthesis. The developmental stages just after cellularization when ETCs have adopted cell fate and first cell wall depositions can be detected (5–7 DAF) is consistent with the idea that the demand for respiratory energy and carbohydrates is high for cell wall development.

### THE PATTERN OF TRANSCRIPTIONALLY ACTIVATED TRANSPORTER GENES CHANGES WITH THE ONSET OF GRAIN FILLING

**FIGURE 2:**
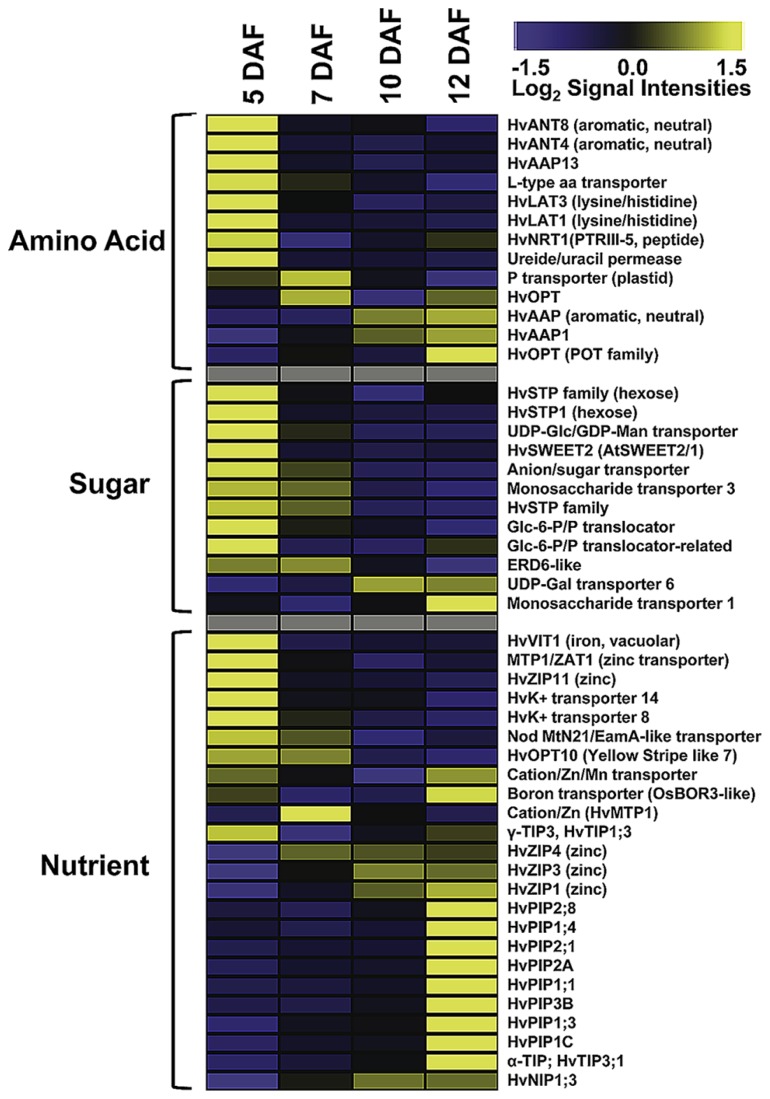
**Expression profiles of transporter genes in developing ETCs as determined by microarray analysis.** Transcript levels are median-centered and log_2_-transformed. Transcript levels are indicated by color code: yellow, high; blue, low [data were selected from transcriptome analysis presented by Thiel et al. (2012a)].

As ETCs control assimilate and nutrient uptake from maternal tissues into the endosperm via an apoplasmic barrier, the transcriptional activities of transporter genes address the main function of these cells. Developing seeds are sink tissues importing photoassimilates in solution by bulk flow through the phloem. Major transported solutes are sucrose, amino acids and monosaccharides. Expression of invertases and monosaccharide transporter in early legume and cereal seed development governs the distribution of free sugars which play a pivotal role in regulating TC function and simultaneously, determining final endosperm and embryo cell number (Thompson et al., 2001). ETC-specific transcriptome analysis at distinct stages of barley grain development showed a wide array of expressed transporter genes possibly related to endosperm development (**Figure 2**). Several genes show a peak of expression directly after cellularization (5 DAF); eight amino acid, nine sugar, and two potassium transporter are highly expressed. Transcriptional activity of several hexose transporters, including *HvSTP1* and transporter for Glc-6-phosphate, UDP-Glc and UDP-galactose, indicate a high influx of hexose sugars probably feeding glycolysis or providing precursors of cell wall synthesis. The high expression of transcripts related to cell wall biosynthesis, particularly to UDP-Glc metabolism corresponds to transporter gene activities. *HvSTP1* has been shown to be expressed in very early endosperm development (from the syncytial stage on) and to be spatially and temporally associated with the cell wall invertase1 (*HvCWIN1*) suggesting an interplay between liberation of hexoses by invertase activity and active uptake by *HvSTP1* (Weschke et al., 2003). An interesting gene is the barley ortholog to the wheat *TaSWEET2*, assigned as *HvSWEET2*, which depicts the same profile with highest transcript levels at 5 DAF. Most of the recently discovered membrane-associated SWEET proteins have been shown to function as monosaccharide, particular glucose transporter in *Arabidopsis* (Slewinsky, 2011). A genetic analysis of *AtSWEET11* and *-12* revealed that these two transporters are capable of transporting sucrose and control sucrose release from phloem parenchyma TCs for uptake into sieve element/companien cell complexes (Chen et al., 2012). A set of amino acid transporter with different substrate specificities (neutral/aromatic, lysine/histidine, peptides; assignment according to Kohl et al., 2012) is predominantly expressed at 5 DAF corresponding to a stimulated amino acid metabolism at this time point. All of these transporters have in common that they catalyze proton-coupled/energy-dependent active transfer of nutrients. In addition to the transcriptome analysis, metabolite profiling of developing ETCs has been performed by a GC–MS approach (Thiel et al., 2012a). Metabolite abundancies display distinct profiles during development from cellularization until further differentiation/maturation of ETCs. Several amino acids, among them the transport form glutamine and lysine, are most abundant at 5 DAF. Enhanced glutamine levels potentially result from high activity of Gln synthase in the NP which has been observed by a tissue-specific metabolite analysis (Thiel et al., 2009). Cells of NP may convert amino acids in transport forms for active uptake from the apoplasmic endosperm cavern by the ETCs. This example nicely demonstrates a coordinated action of these transfer-related tissues at the maternal/filial boundary in allocation of nitrogen to the endosperm. Hexose sugars, like fructose, glucose and glucose-6-phosphate show the highest concentrations at 5 and 7 DAF, respectively, and thereby correspond to the pronounced activities of monosaccharide transporter genes. Sucrose levels clearly peak at 7 DAF and may be an indicator for the switch from high to low hexose:sucrose ratios which might serve as an intracellular signal for the transition of the endosperm into a storage accumulating organ. A recent comparative transcriptome analysis of cells of NP and ETCs in barley grains was in line with these presumptions and revealed a pronounced expression of *HVSUT1* and two amino acid permeases (AAPs) in ETCs before the beginning of grain filling (Thiel et al., 2008).

As visible in **Figure 2**, the spectrum of transcriptionally activated transporter genes clearly changes with the onset of the storage phase (10 DAF). Nutrient transporter with less defined substrate specificities, such as different members of the aquaporin family, and micronutrient transporter displayed a strong increase of transcript amounts at 12 DAF, whereas the expression of sugar and amino acid transporter significantly decreased. Aquaporins are channel proteins positioned in the plasma and intracellular membranes of plant cells and function as facilitators of water, neutral solute or gas transport. Aquaporins in plants can be classified into plasma membrane intrinsic proteins (PIPs), tonoplast intrinsic proteins (TIPs), NOD26-like proteins (NIPs), and small basic intrinsic proteins (SIPs). Several HvPIP isoforms specifically peak at 12 DAF and might regulate water and solute flow from the endosperm cavity into the endosperm. Different members of the PIP and TIP family have been shown to be transcriptionally activated in the seed coat and cotyledons of pea seeds and a role in nutrient release for the embryo is anticipated (Schuurmans et al., 2003). A general role in sensing the osmotic potential and regulation of turgor homeostasis necessary for water equilibrium between phloem and xylem is assumed for PIP aquaporins (Zhang et al., 2007). TIP isoforms are probably localized to different vacuolar compartments from which the subgroup of α-TIPs is postulated to be predominantly associated to protein storage vacuoles (Jauh et al., 1999) and has been found exclusively in developing embryos (Hunter et al., 2007) pinpointing to a role in lytic/remobilization processes. Noticeable is the emerging expression of three genes encoding zinc-/iron-regulated transporter-like proteins (ZIPs), cation/Zn transporter/metal tolerance protein (HTP1) and a boron transporter from 10 DAF on. ZIPs are capable of transporting divalent metal ions (i.e., zinc, iron) and are suggested to play critical roles in balancing metal uptake and homeostasis. Homeostasis of micronutrients is essential for plant growth and development as their deficiency or excess severely impaired physiological and biochemical reactions of plants. The storage of micronutrients in seeds is of high relevance for seedling growth after germination and represents also an important trait for the nutritional value of crop seeds. In maize kernels, novel identified ZmZIP genes revealed distinct temporal expression patterns in embryo and endosperm indicating a complex regulation of ion translocation and/or storage in embryo and endosperm development (Li et al., 2013). A transcriptome analysis of different tissues from barley grains observed specific patterns of metal transporter activities in embryo, endosperm, aleurone, and maternal tissues of the transfer zone (i.e., NP cells; Tauris et al., 2009). Comparable to ETCs (see **Figure 2**), members of the ZIP, VIT, and yellow stripe like (YSL) families have been shown to be highly expressed in the transfer zone and probably contribute to zinc/metal supply to the endosperm and/or embryo, but due to different developmental time points of the investigated grains (onset of storage phase – late storage phase) a direct relation is difficult to draw. A study visualizing nutrient distribution in the wheat grain at the end of the storage phase (25 DAF) by laser ablation inductively coupled plasma mass spectrometry (LA-ICP-MS) imaging showed an accumulation of micronutrients preferentially in the scutellum of the embryo (Wu et al., 2013). Besides, iron and zinc seem to be enriched in the ETC region but with lower amounts compared to the scutellum. Accordingly, a transfer route of these micronutrients for provision of the embryo could start with an uptake from the maternal sites of efflux (NP) in the filial grain part by ETCs, from where it is directed toward the embryo-near region and actively absorbed by the scutellum. Assuming the high expression of ZIPs at the onset of the storage phase (10 DAF) this developmental stage could represent the starting point for Zn/Fe-accumulation in the embryo, although micronutrient distribution patterns have been analyzed later in grain development and need to be verified for earlier stages.

In conclusion, transcript profiles of transporter genes in ETCs revealed development-specific patterns of expression. Although transcript abundancies do not necessarily reflect protein amounts or temporal protein activities, the data suggest a general transition from active to passive modes of transport. After cellularization and during early ETC development (5–7 DAF) active nutrient uptake seems to be dominating as concluded from high transcript amounts of amino acid, monosaccharide and potassium transporter, which are energy coupled and driven by the proton-motive force. High hexose and amino acid concentrations, which have been shown to peak between 5 and 7 DAF, are probably needed for differentiation processes in the ETCs itself but also for cell proliferation and rapid growth of the endosperm. Between 7 and 10 DAF a change in gene activities from active to passive transport mechanisms was observed. Expression of micronutrient transporter and an array of aquaporins arose with the beginning of the storage phase. In accordance, intracellular changes have been observed in ETCs at 12 DAF. The cytoplasm of ETCs seems to be generally reduced compared to earlier stages, possibly this structural modifications might be associated with the shift to passive modes of transport. In summary, observed changes might be related to the switch in caryopsis development during the intemediate phase between 6 and 10 DAF where a metabolic and transcriptional reconfiguration indicates the transition of the endosperm into a storage accumulating organ (Sreenivasulu et al., 2006). The beginning of the accumulation process coincides with the switch from maternal to filial control of grain development and this might be also reflected in the changing patterns of transporter gene activity.

## IDENTIFICATION OF SIGNALS REGULATING ENDOSPERM TRANSFER CELL SPECIFICATION AND DIFFERENTIATION

### TISSUE-SPECIFIC 454 TRANSCRIPTOME SEQUENCING IDENTIFIED TCS PHOSPHORELAYS AS A MAJOR SIGNAL TRANSDUCTION PATHWAY IN ETCs

**FIGURE 3:**
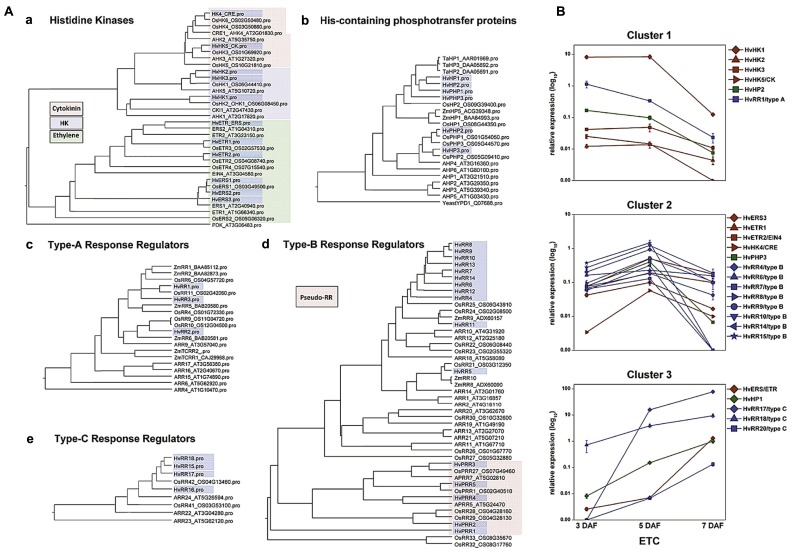
**Phylogenetic relationship of TCS elements from barley, *Arabidopsis* and rice and expression profiles of selected genes in barley ETCs.** **(A)** Barley sequences are highlighted by blue boxes. **(a)** Histidine kinases, colors indicate different subgroups. *Arabidopsis* PDK was used as outgroup. **(b)** HPt elements, protein sequences of *Z. mays* and *T. aestivum* were additionally included in the alignment. Yeast HPt protein YPD1 was used as outgroup. **(c–e)** Type-A, -B, -C response regulators, amino acid sequences of selected maize response regulators were included in the alignment. **(B)** Expression profiles of barley TCS elements in ETCs as determined by qRT-PCR analyses. Expression profiles of candidate genes were grouped by clusters. [Images are reproduced from Thiel et al. (2012b) with permission of PLoS org.].

To identify new sequence information putatively involved in early differentiation of barley ETCS, 454 transcriptome sequencing of the differentiating ETC region from the syncytial stage until functionality has been performed (3–7 DAF). Tissues were isolated by laser-assisted microdissection and the transcriptome was analyzed by pyro-sequencing. Surprisingly, about 40% (17,028) of the generated contigs were not present in barley EST databases and represent ETC-specific sequences (Thiel et al., 2012b). Screening the data set for signaling components uncovered an intriguingly high amount of transcripts encoding elements of the two-component signaling (TCS) system to be specifically expressed in this endosperm region. Fourty genes probably being part of the TCS were identified in the ETC transcriptome during the narrow time period of 4 days, thereby covering all components and subfamilies of the TCS system (**Figure 3A**). The TCS represents an ancient signal transduction pathway firstly discovered in bacteria and has previously been shown to be co-opted by eukaryotic organisms, like fungi and plants, whereas in animals and humans this signaling route does not exist. In plants, the TCS persists of a multi-step phosphorelay involving phosphorylation of His and Asp residues of proteins in a modular arrangement and thereby, transmits a signal from the membrane to the nucleus to modulate cellular responses (Schaller et al., 2008). TCS phosphorelays have been shown to be implicated in the perception of external signals, such as sensing of nutrient availability, abiotic stresses, and to regulate various aspects of plant development (Sakakibara et al., 1998; Leibfried et al., 2005; Mizuno, 2005; Chefdor et al., 2006; Kim et al., 2006; Riefler et al., 2006). Information about implications in developmental programs of plants is largely related to cytokinin signaling (Schaller et al., 2008), but interactions with signaling pathways of other hormones, like ethylene, abscisic acid (ABA) or auxins, are indicated by several studies (Hass et al., 2004; Wohlbach et al., 2008; Kakani and Peng, 2011; Scharein and Groth, 2011).

As presented in the phylogenetic tree in **Figure 3Aa** three types of histidine kinase (HK) transcripts were found in barley ETCs: six putative ethylene receptors characterized by a GAF domain, two putative cytokinin receptors with an emblematic CHASE domain and three classical HKs. Six genes encode histidine-containing phosphotransfer proteins (HPts) which act as intermediates in multi-step phosphorelays by converting signals from HKs to response regulators. Among them, three elements with conserved His residues and three pseudo-HPts are transcriptionally activated in ETCs. Response regulators (RRs) execute the signal output of the signal transduction pathway by activating downstream target genes. Three subclasses of RRs have been identified in the ETC transcriptome: three type-A and four type-C RRs which are solely composed of a receiver (REC) domain with short N- and C-terminal extensions and 11 sequences encoding B-type RR elements (Thiel et al., 2012b). Type-B RRs are structurally differing from other RRs as they contain long C-terminal extensions with a MYB-like DNA binding domain, suggesting that they act themselves as transcription factors (Imamura et al., 1999). The fact that to a great extent TCS sequences were not present in public EST databases supports the specificity for small distinct tissue regions, like differentiating ETCs, and subsequently, indicates an outstanding role for the TCS in intrinsic developmental programs of these cells.

Pioneering work toward a role for TCS phosphorelays in differentiation of maize BETCs came from Muñiz et al. (2006, 2010). They showed a specific transcript accumulation of *ZmTCRR1* and *-2* in the BETL layer and verified a physical interaction between both proteins with the phospho-intermediate ZmHP2. By qRT-PCR analysis other elements putatively contributing to TCS phosphorelays in maize kernels have been identified but a specific localization of these TCS elements in BETCs has not been resolved so far. Transcript profiling of barley TCS elements in isolated ETCs by qRT-PCR identified clusters of co-expressed genes activated in a consecutive manner during development (**Figure 3B**). At 3 DAF just at the transition from the syncytial state to cellularization, the HK *HvHK1* is highly and exclusively expressed in the ETC region. Concomitantly HvHP2 and the type-A response regulator HvRR1 show an overlapping profile with a truncated expression at 5/7 DAF (cluster 1). After cellularization when cells have adopted ETC fate, several putative ethylene receptors, a HPt element (HvPHP3) and a suite of type-B RRs depict a characteristic peak of expression at 5 DAF (cluster 2). During progression of structural specification of ETCs another putative ethylene receptor is coordinatively expressed with HvHP1 and two type-C RRs (HvRR15/-16), which both depict outstandingly high transcript levels (cluster 3). Assuming that genes sharing a common function and/or a functional relation are frequently transcriptionally co-ordinated, co-expression analysis of TCS elements pinpoints to a participation in specific phosphorelays activated at important crossroads of ETC development. The experimental design of analysis also provided information about the specificity of gene expression in ETCs related to other grain tissues: transcripts assigned to clusters 1 and 2 were almost found to be exclusively expressed in ETCs whereas transcripts of cluster 3 also depict a remarkable expression in other tissues of the grain (see Thiel et al., 2012b).

### A CROSSTALK OF SPECIFIC TCS ELEMENTS AND ABA MIGHT REGULATE ETC CELLULARIZATION

**FIGURE 4:**
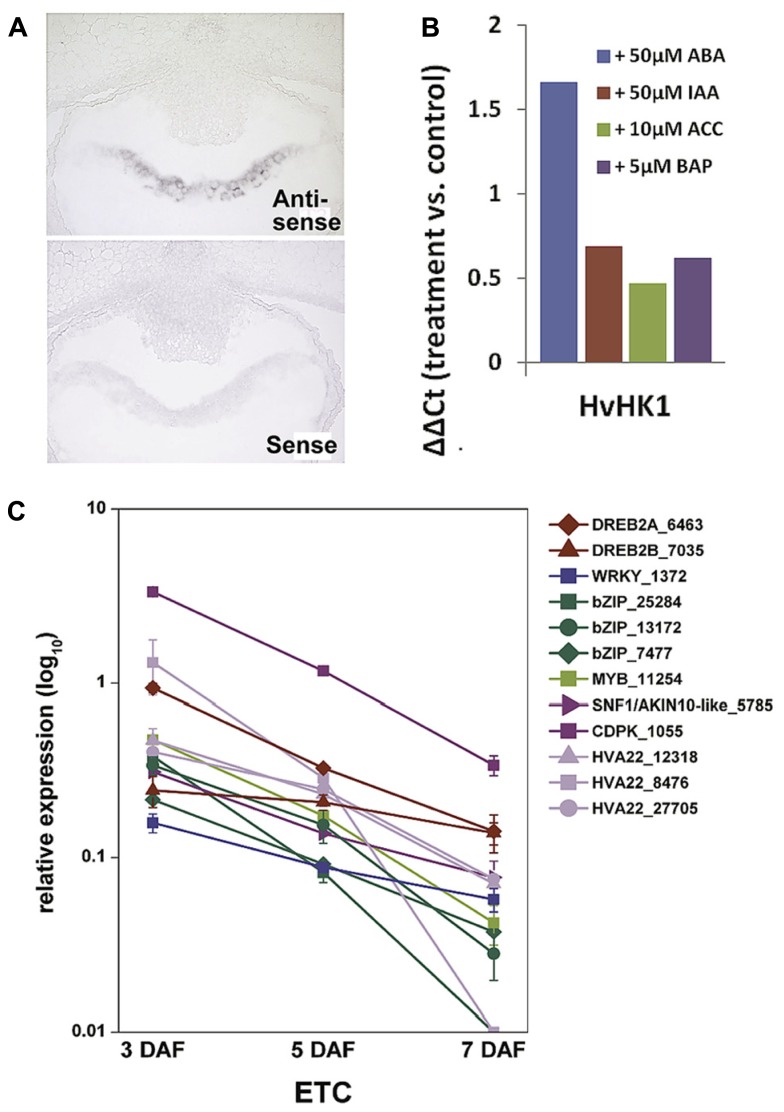
**Transcript analyses of *HvHK1* and putatively interacting transcription factors.** **(A)** *In situ* hybridization specifies the expression of *HvHK1* in 3 DAF grains exclusively in the part of the cellularizing endosperm facing the nucellar projection. **(B)** Transcriptional response of *HvHK1* to hormones was analyzed by *in vitro* experiments. Grains were incubated in MS medium supplemented with the indicated concentrations of hormones for 16h. The ΔΔCt value was calculated from qRT-PCR analysis and represents the log_2_-ratio compared to control conditions. **(C)** Expression profiles of putative interacting transcription factors and ABA signaling elements in ETCs. Transcript levels were determined by qRT-PCR analyses and relative expression is given in the log_10_ scale. Transcripts numbers indicate the contig identifier in the 454 transcriptome assembly [Image in C is reproduced from Thiel et al. (2012b)].

Membrane-bound HKs are expected to play a key role in TCS-mediated signal transduction pathways as they perceive extracellular signals which are finally transmitted via the cytoplasm into the nucleus. The deciphered localization of such a receptor component at the exchange surface of maternal and filial tissues generally hints at a role in signal transmission between the mother plant and the filial tissues. Among the TCS elements transcriptionally activated in the ETC region before cellularization (see **Figure 3B**), a decisive role is anticipated for *HvHK1*, which is the only HK remarkably been expressed in the syncytial endosperm. *In situ* hybridization verified the expression of *HvHK1* at 3 DAF exclusively in the part of the syncytium/cellularizing endosperm facing NP (**Figure 4A**). Interestingly, in the phylogenetic tree *HvHK1* clusters together with the *Arabidopsis* receptors AHK1 and Cytokinin Independent 1 (CKI1; **Figure 3A**). Genevestigator data show an expression of *AHK1* and *CKI1* in the chalazal endosperm (Zimmermann et al., 2004), which is one of the mitotic domains in the early syncytial endosperm positioned at the maternal-filial boundary of *Arabidopsis* seeds. In analogy to the ETC region, the chalazal endosperm facilitates nutrient transfer from the mother plant to the next generation (Boisnard-Lorig et al., 2001). AHK1 was initially identified as a plant osmosensor and thereby acting as a positive regulator of salt and drought stress responses (Tran et al., 2007). By using single T-DNA insertion lines and AHK1 overexpression, AHK1 has previously been shown to positively affect ABA signaling and to enhance ABA biosynthesis in vegetative and seed tissues (Wohlbach et al., 2008). By *in vitro* incubation experiments with barley grains (4 DAF), a strong transcriptional response of *HvHK1* to external ABA was monitored; transcript levels increased to more than 3-fold (**Figure 4B**). To check if there are additional hints for ABA-mediated transcriptional regulation, we searched for conserved *cis*-regulatory motifs in the promoter regions of *HvHK1* and co-expressed TCS elements *HvHP2* and *HvRR1*. As now comprehensive information about the barley genome is available (http://webblast.ipk-gatersleben.de/barley/viroblast.php) promoter sequences 1 kb upstream of the predicted start codon were identified and analyzed for the occurrence of transcription factor binding sites. Promoter motifs known to be bound by transcription factors implicated in ABA signaling (ATHB, ABRE, DREB, MYC, MYB, and W-box) are highly abundant in the investigated promoter regions. The co-occurrence of *cis*-elements potentially interacting with ABA-related transcription factors reveals common mechanisms of transcriptional regulation by ABA (Thiel et al., 2012b). To encourage the role of putative interacting transcription factors, the ETC-specific 454 contigs were screened for candidate genes from these putative interaction partners. Transcript profiling by qRT-PCR revealed that three putative bZIP transcription factors, two putative DREB transcription factors (DREB2A, DREB2B), a MYB and a WRKY transcription factor, are highly expressed at 3 DAF (**Figure 4C**). Furthermore, three members of the HVA22 family, genes which are strongly induced by ABA (Shen et al., 2001), as well as candidate genes operating as positive regulators on the ABA signaling pathway (CDPK, SNF1-related kinase) depict the same profile. In summary, profiles of ABA signaling elements and transcription factors support the idea that ABA regulates transcriptional networks involved in ETC cellularization in a crosstalk with the phosphorelay genes *HvHK1, HvHP2, and HvRR1*. Protein-DNA binding studies would be helpful to verify transactivation of the phosphorelay genes by ABA-related transcription factors as suggested by the results. The identification of transcription factors transcriptionally activating phosphorelay genes with a proposed role in ETC specification would expand the knowledge about ABA-regulated signaling cascades in a fundamentally important process of seed development. The idea that ABA affects early endosperm development of seeds is supported by other studies in *Arabidopsis* and rice. Transcriptome analyses of the developing rice endosperm (Xue et al., 2012) and the proliferating *Arabidopsis* endosperm at the syncytial stage (Day et al., 2008) revealed a pronounced expression of ABA biosynthesis and signaling genes during early endosperm differentiation. The studies correspond to results obtained from the analysis of the barley endosperm mutant *seg8* for which defects in cellularization of ETCs have been attributed to altered ABA levels (Sreenivasulu et al., 2010). The study implies a role for ABA in cell cycle regulation during early endosperm development. Related to the function of potentially ABA-regulated TCS elements, which expression disappears after ETC cellularization, a possible role for the maintenance of the coenocyte and/or the switch to cellularization can be concluded for *HvHK1, HvHP2, and HvRR1*.

### PHOSPHORELAYS MIGHT BE INVOLVED IN ETHYLENE SIGNAL TRANSDUCTION AND REGULATE ESTABLISHMENT OF TRANSFER CELL STRUCTURE

Information about implications of the TCS in developmental programs of plants is largely related to cytokinin signaling in *Arabidopsis* (Schaller et al., 2008). However, elements of a subgroup of HKs (ETR/ERS) have been identified as ethylene receptors and negative regulators of the ethylene response, but the contribution of downstream phosphorelay elements in ethylene signal transduction has been discussed controversially. Previous results hinted at an implication of specific HPt and RR elements in ethylene signal transduction. Urao et al. (2000) detected physical interactions between the ethylene receptor ETR1 and three HPt intermediates as well as interactions of them with type-A response regulators. By genetic analysis, it was concluded that type-B response regulator ARR2 mediates ethylene responses and its transcription factor activity is likely to be regulated by an ETR1-initiated phosphorelay (Hass et al., 2004). More recently, an interaction of ETR1 with the HPt protein AHP1 has been evidenced by fluorescence polarization. Using this method the authors additionally confirmed that the affinity of the ETR1-AHP1 complex is tightly regulated by ethylene (Scharein and Groth, 2011). qRT-PCR profiling of TCS elements in ETCs revealed transcriptional activation of different putative ethylene receptors with concomitant downstream elements after cellularization (clusters 2 and 3, **Figure 3B**). Data of co-expressed TCS elements in barley ETCs between 5 and 7 DAF allow the assumption that type-B and type-C RRs, respectively, participate in ethylene receptor-initiated phosphorelays. An interesting fact is that the MYB-related transcription factor ZmMRP-1 (Gomez et al., 2009) and just lately, two MYB-related genes (Chinnappa et al., 2013) have been identified as transcriptional “master switches” regulating wall ingrowth deposition in BETCs of maize kernels and phloem parenchyma TCs in *Arabidopsis*, respectively. The high number of barley type-B RRs with a MYB-like DNA binding domain and their specific expression in ETCs after cellularization (5 DAF) imply a role for MYB domain-containing proteins as regulatory components in transcriptional networks controlling wall ingrowth development.

Supporting data for a key role of ethylene in barley ETC development came from microarray analysis of developing ETCs. High transcriptional activity of the key enzymes in ethylene biosynthesis, *S*-adenosylmethionine (SAM) synthase and ACC oxidase, particularly at 5 DAF indicates a burst in ethylene production after cellularization of ETCs, which is followed by an upregulation of ethylene signaling elements at 7 DAF or later stages. Confirmed players in ethylene signal transduction are ethylene receptors, ethylene insensitive 3 (EIN3) and EIL (EIN3-like) transcription factors operating upstream of ERF1/EREBP1 transcription factors. The combination of metabolite analysis with transcriptional activities gave further indications for an activated methylation (Yang) cycle in ETCs, which might provide the ethylene precursor SAM. Pronounced expression of genes encoding enzymes involved in methionine and SAM recycling correlate to changing levels of the metabolic intermediates cysteine, isoleucine, and methionine between 5 and 10 DAF (Thiel et al., 2012a). Considering the findings that ethylene signaling directs initiation of TC morphology in *V. faba* cotyledons and application of ACC induces formation of wall ingrowths in tomato roots (Schikora and Schmidt, 2002; Dibley et al., 2009), high expression of ethylene biosynthesis and signaling genes in barley ETCs hints at an implication of ethylene in the formation of wall ingrowths. An important question is whether the downstream phosphorelay elements (HvHPts and HvRRs from B-and C-type) co-regulated with HvETR/ERS elements are part of ethylene receptor-initiated phosphorelays and/or if they interact with downstream elements in ethylene signal transduction. Protein-protein interaction studies could confirm specific interactions between TCS elements in ethylene receptor-initiated phosphorelays and known ethylene signaling elements as suggested by co-expression analysis.

## CONCLUSIONS AND FUTURE DIRECTIONS

**FIGURE 5:**
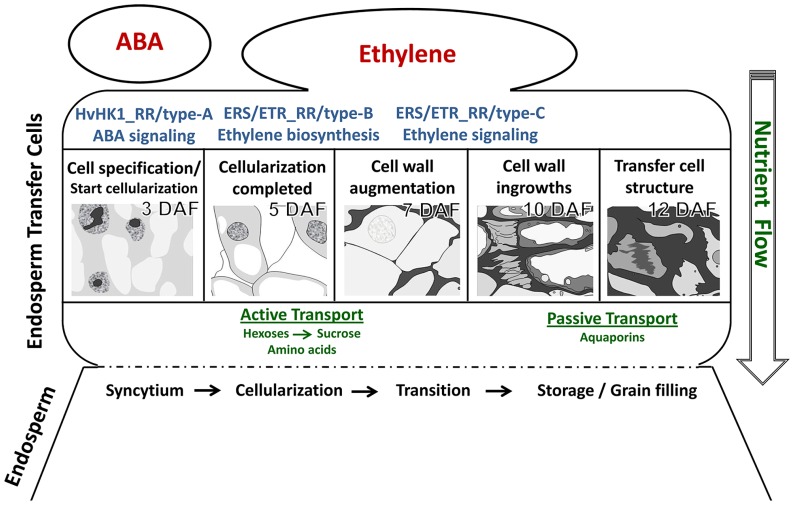
**Hypothetical model of cellular processes and signals determining ETC differentiation from cell specification to the establishment of transfer cell morphology.** The figure summarizes results from different kinds of tissue-specific analyses. Further explanations are given in the text.

Recent technological developments in providing access to specific cellular regions or even single cells have advanced our understanding of differentiation processes occurring inside an organ or in specific tissues. LM-based methods have brought knowledge gains about developmental programs involved in differentiation processes of ETCs in barley grains. Insights into ETC differentiation as discussed in this review are schematically represented in **Figure 5**. Global transcriptome analysis highlighted activated pathways in cellular differentiation and cell wall metabolism which could be related to transport mechanisms (active→passive) into the endosperm. A decisive role for ABA in ETC specification and for ethylene as an inductive compound for cell wall modification was hypothesized. Expression of ABA-related transcription factors and signaling elements just at the onset of cellularization as well as transcriptional regulation of *HvHK1* pinpoints to early ABA influences on cell specification which might be perceived by *HvHK1*. Ethylene biosynthesis is clearly stimulated in ETCs after cellularization (5 DAF) in correspondence to transcriptionally activated ethylene receptor-initiated phosphorelays. Potential ERS/ETR-phosphorelays involve type-B RRs at 5 DAF whereas during further ETC differentiation other RR elements (type-C subgroup) might contribute to ethylene signaling pathways. Changing phosphorelay elements during the establishment of TC anatomy is accompanied by the upregulation of downstream elements on the ethylene signal transduction pathway. Accordingly, a cascade of ethylene biosynthesis in ETCs and successive ethylene regulation might be responsible for structural modifications of ETCs which in turn are linked to altered modes of transport.

The discovery of signals determining ETC differentiation in barley grains also represents the starting point for further investigations. Functional studies *in planta* by modulating the expression of identified candidate genes will help to advance our understanding about the importance of TCS pathways for ETC and endosperm development. The verification of postulated interactions between phosphorelay elements and putative intersections with ABA and ethylene signaling pathways would emerge new aspects of hormonal regulation in crop seed development.

## Conflict of Interest Statement

The author declares that the research was conducted in the absence of any commercial or financial relationships that could be construed as a potential conflict of interest.
